# The Peptide PnPP-19, a Spider Toxin Derivative, Activates μ-Opioid Receptors and Modulates Calcium Channels

**DOI:** 10.3390/toxins10010043

**Published:** 2018-01-15

**Authors:** Ana C. N. Freitas, Steve Peigneur, Flávio H. P. Macedo, José E. Menezes-Filho, Paul Millns, Liciane F. Medeiros, Maria A. Arruda, Jader Cruz, Nicholas D. Holliday, Jan Tytgat, Gareth Hathway, Maria E. de Lima

**Affiliations:** 1 Departamento de Bioquímica e Imunologia, Universidade Federal de Minas Gerais, Belo Horizonte 31270-901, Brazil; acnfreitas@gmail.0100com (A.C.N.F.); flavio.hpmacedo@gmail.com (F.H.P.M.); menezesfilho10@gmail.com (J.E.M.-F.); jadercruzytrio@gmail.com (J.C.); 2 Toxicology and Pharmacology, KU Leuven, 3000 Leuven, Belgium; steve.peigneur@pharm.kuleuven.be (S.P.); jan.tytgat@pharm.kuleuven.be (J.T.); 3 Arthritis Research UK Pain Centre, School of Life Sciences, Queen’s Medical Centre, University of Nottingham, Nottingham NG7 2UH, UK; paul.millns@nottingham.ac.uk (P.M.); gareth.hathway@nottingham.ac.uk (G.H.); 4 Cell Signaling Research Group, School of Life Sciences, Queen’s Medical Centre, University of Nottingham, Nottingham NG7 2UH, UK; licimedeiros@gmail.com (L.F.M.); Maria.Arruda@nottingham.ac.uk (M.A.A.); nicholas.holliday@nottingham.ac.uk (N.D.H.); 5 Farmanguinhos, Fiocruz, Brazilian Ministry of Health, Rio de Janeiro 22775-903, Brazil

**Keywords:** *Phoneutria nigriventer*, opioid receptor, spider toxin, antinociception, The spider toxin derivative PnPP-19 activates μ-opioid receptors and blocks calcium channels in DRG neurons. Our data highlights the possible use of PnPP-19 for the development of new drug candidates for pain treatment.

## Abstract

The synthetic peptide PnPP-19 comprehends 19 amino acid residues and it represents part of the primary structure of the toxin δ-CNTX-Pn1c (PnTx2-6), isolated from the venom of the spider *Phoneutria nigriventer*. Behavioural tests suggest that PnPP-19 induces antinociception by activation of CB1, μ and δ opioid receptors. Since the peripheral and central antinociception induced by PnPP-19 involves opioid activation, the aim of this work was to identify whether this synthetic peptide could directly activate opioid receptors and investigate the subtype selectivity for μ-, δ- and/or κ-opioid receptors. Furthermore, we also studied the modulation of calcium influx driven by PnPP-19 in dorsal root ganglion neurons, and analyzed whether this modulation was opioid-mediated. PnPP-19 selectively activates μ-opioid receptors inducing indirectly inhibition of calcium channels and hereby impairing calcium influx in dorsal root ganglion (DRG) neurons. Interestingly, notwithstanding the activation of opioid receptors, PnPP-19 does not induce β-arrestin2 recruitment. PnPP-19 is the first spider toxin derivative that, among opioid receptors, selectively activates μ-opioid receptors. The lack of β-arrestin2 recruitment highlights its potential for the design of new improved opioid agonists.

## 1. Introduction

The venom of *Phoneutria nigriventer* has been the focus of intensive research in recent years since it is of interest for discovering novel pharmaceutical bioactive peptides. This venom has a potent neurotoxic effect and many of its toxins have been already isolated and studied in detail [1]. One of the best characterized toxins, δ-CNTX-Pn1c, also known as PnTx2-6 [2], exerts interesting pharmacological effects and it was originally studied as a modulator of voltage-gated sodium channels [1]. Recently, this toxin has been studied as a potentiator of erectile function. δ-CNTX-Pn1c improves erectile function of normotensive and DOCA-salt hypertensive rats [3] and it also ameliorates the erectile function of rats with bilateral cavernous nerve crush injury [4].

The synthetic peptide PnPP-19 comprehends 19 amino acid residues and it represents part of the primary structure of the spider toxin δ-CNTX-Pn1c. This peptide has been suggested to be a promising drug candidate for the treatment of both erectile dysfunction and pain. Through histopathological experiments [5], it was shown that PnPP-19 does not induce any sign of toxicity in different tissues (brain, heart, lung, liver and kidney) and it no longer modulates Nav channels [5]. Furthermore, it does not cause death or hypersensitivity reactions and it induces only low immunogenicity in mice. However, similar to the native toxin δ-CNTX-Pn1c, PnPP-19 does potentiate erectile function. The exact molecular target through which PnPP-19 improves erectile function still awaits elucidation [5]. Regarding the pain pathway, PnPP-19 induces both peripheral and central antinociception. This antinociceptive effect elicited by the peptide seems to involve the activation of opioid and cannabinoid receptors along with the activation of the NO/cGMP/K_ATP_ pathway [6,7,8].

Millions of people suffer from acute or chronic pain every year, which makes pain a serious global public health problem. Chronic pain, for instance, may cause an enormous socioeconomic impact with associated costs in treatment and reduced levels of productivity [9]. Nowadays, there is an urge for the development of novel potent and more selective analgesic drugs that elicit less undesirable side effects [10]. 

The opioid receptors belong to the superfamily of GPCRs and they are coupled to G_i_/G_o_ proteins. The activation of these receptors may contribute to cellular hyperpolarization, and might impair neurotransmitters release, by suppressing calcium influx and stimulating potassium channels. The activation of the three different opioid receptor subtypes (μ-, δ- and κ-) might inhibit different calcium channels in various mammalian tissues [11]. Therefore, measuring calcium currents could be a complementary way for verifying opioid activation. In addition, the direct inhibition of calcium channels by exogenous substances may also induce per se antinociception. This is the case, for example, of two antinociceptive *P. nigriventer* toxins, PnTx3-3 and PnTx3-6 [12,13], and the well-known Food and Drug Administration (FDA) approved analgesic drug Prialt^®^ (Ziconotide) [14]. Regarding the potassium channels, it has been shown that opioid receptor activation leads to opening of different potassium channels, among which are inward rectifying potassium channels (GIRK) [11]. As such, measuring the alteration of potassium flux through the cell membrane might also be an alternative way of investigating opioid receptor activation.

Since the peripheral and central antinociception induced by PnPP-19 involves opioid activation [6,8], the aim of this work was to identify whether this synthetic peptide could directly activate opioid receptors and investigate the possible subtype selectivity for μ-, δ- and/or κ-opioid receptors co-expressed with GIRK1/GIRK2 and RGS4. Furthermore, we also studied the modulation of calcium influx driven by PnPP-19 in dorsal root ganglion (DRG) neurons, and analyzed whether this modulation was opioid-mediated. Our data show that PnPP-19 may selectively activate μ-opioid receptors, however with low potency. Interestingly, activation of opioid receptors induced by the PnPP-19 does not stimulate the recruitment of β-arrestin2. However, it does induce indirectly inhibition of calcium channels and, consequently, impairs calcium influx in DRG neurons.

## 2. Results

### 2.1. Electrophysiological Characterization of Direct Activation of Opioid Receptors Induced by PnPP-19 Using Two-Electrode Voltage-Clamp

**Figure 1:**
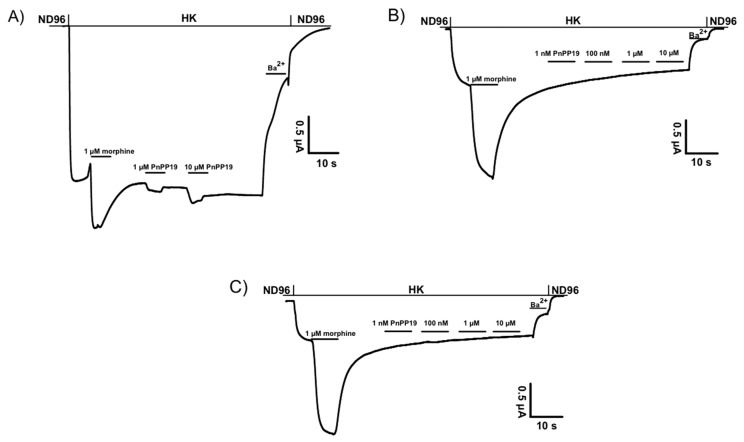
(**A**) Shows representative current traces of agonist-gated currents evoked from oocytes expressing human μ-opioid receptor (hMOR) by 1 μM morphine and 1 or 10 μM PnPP-19. PnPP-19 could not activate human δ-opioid receptor (hDOR) (**B**) or human κ-opioid receptor (hKOR) (**C**) up to a concentration of 10 μM.

Each receptor was individually co-expressed with GIRK1/GIRK2 channels and RGS4, mimicking the native neuronal G-protein-mediated pathway of K^+^ channel activation. We used the two-microelectrode voltage-clamp technique to measure the opioid receptor-activated GIRK1/GIRK2 channel response as the increase of the inward K^+^ current at −70 mV, evoked by the application of increasing concentrations of opioid ligands. The potency of PnPP-19 on human μ-opioid receptor (hMOR), human κ-opioid receptor (hKOR) and human δ-opioid receptor (hDOR) was investigated (Figure 1). Concentrations up to 10 μM could not evoke currents from oocytes expressing hKOR or hDOR. However, PnPP-19 could activate hMOR, albeit with low potency. Oocytes co-expressing only GIRK1/GIRK2 and RGS4 were used as a control to verify that PnPP-19 indeed interact with the opioid receptor and not the inward rectifying potassium channels. No activity was seen when PnPP-19 was applied to oocytes expressing only GIRK channels and RGS4 (Figure S1).

**Figure 2:**
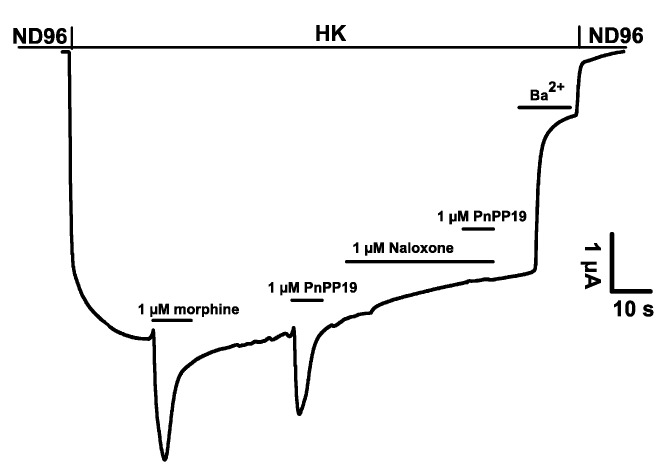
Representative current traces evoked from *X. laevis* oocytes co-expressing GIRK1/GIRK2 channels and RGS4 with hMOR. In addition, 1 μM naloxone inhibits the agonistic activity of PnPP-19.

To confirm the interaction of PnPP-19 with the μ-opioid receptor, the activity of PnPP-19 in the presence of naloxone was investigated (Figure 2). First, expression of GIRK1/GIRK2/RGS4/hMOR was verified by applying 1 μM morphine as a control. Next, 1 μM PnPP-19 was applied as reference current. Application of 1 μM of the well characterized opioid antagonist naloxone was subsequently followed by another pulse of 1 μM PnPP-19. No PnPP-19 evoked current could be observed in the presence of naloxone (Figure 2). A similar experiment, investigating the activation of hMOR by 1 μM morphine in the presence of naloxone was performed as a control (Figure S2).

### 2.2. Inhibition of Calcium Current Induced by PnPP-19

**Figure 3:**
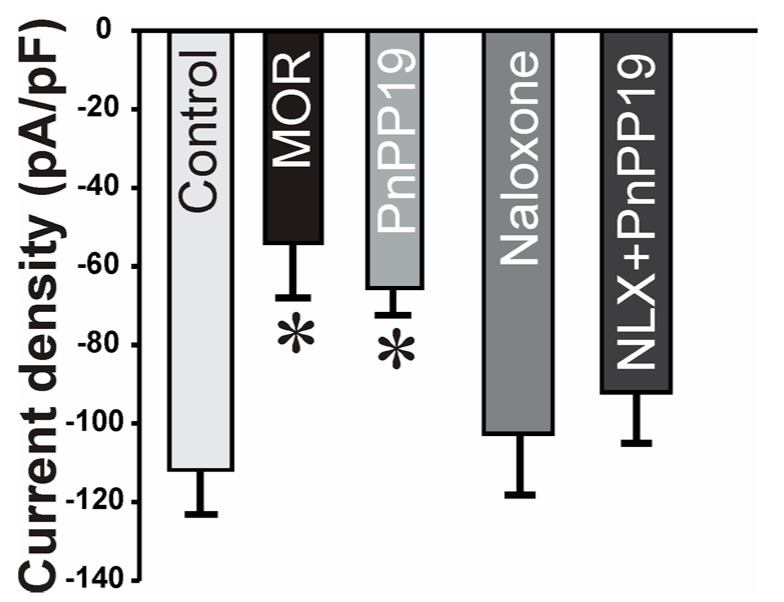
Effect of PnPP-19 and morphine on calcium current density evoked in dorsal root ganglion (DRG) neurons. Calcium currents were evoked by depolarizing pulses to 10 mV (200 ms) from a holding potential of −90 mV in DRG neurons incubated with 1 μM morphine, 1 μM PnPP-19 or 10 μM naloxone. Control group: cells incubated only with external/bath solution. Group NLX + PnPP-19: cells were previously incubated for 30 min with 10 μM naloxone and then PnPP-19 was added reaching a final concentration of 1 μM. MOR: morphine and NLX: naloxone. Data shown are the means ± SEM (*n* = 8 cells, 5 animals). * *p* < 0.05 compared with control (one-way ANOVA + Bonferroni’s test).

Intact neurons of rat dorsal root ganglia (DRG) were used for whole-cell patch-clamp recordings. PnPP-19, morphine or naloxone were added separately to the bath solution to give a final concentration of 1 μM, 1 μM and 10 μM, respectively. Figure 3 shows that PnPP-19 induced a reduction of the calcium evoked current density, with an efficacy comparable to morphine (MOR). Therefore, we could demonstrate that incubation of DRG neurons with the opioid agonist morphine (1 μM) or with PnPP-19 (1 μM) induced inhibition of calcium channels. Furthermore, pre-incubation of the cells with naloxone (10 μM) completely blocks the activity of PnPP-19, suggesting that the inhibition of calcium channels induced by the synthetic peptide is through activation of opioid receptors. Application of naloxone alone has no significant effect on the current density.

### 2.3. Inhibition of Calcium Influx Induced by PnPP-19

**Figure 4:**
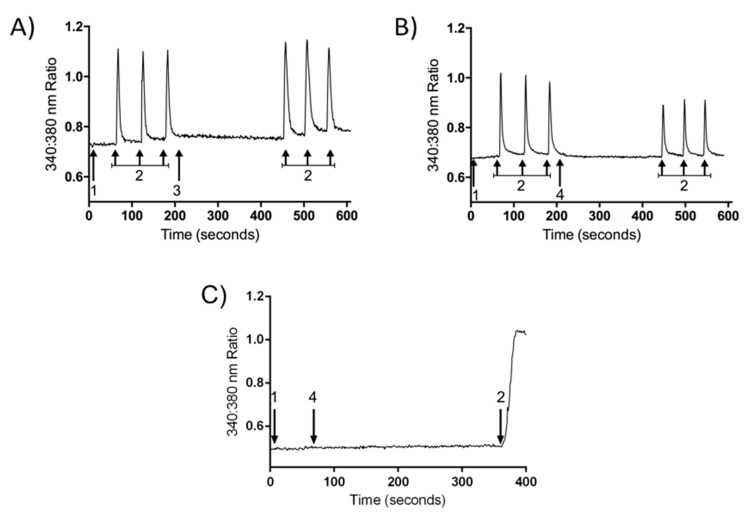
Representative trace showing calcium influx (changes in 340:380 nm ratios) in a single DRG neuron stimulated by KCl before and after 5 min incubation with buffer or PnPP-19. A perfusion system was used to incubate DRG neurons with buffer for 1 min (1) followed by consecutive KCl (30 mM) stimulations (2). After that, cells were perfused with buffer (3) or PnPP-19 (1 μM) (4) for 5 min, and again depolarized by KCl (30 mM) at three different time points (2). Control cells incubated only with buffer after first set of KCl stimulations do not show a significant difference in calcium influx during the second set of stimulations (**A**); However, cells incubated with PnPP-19 display a decrease in calcium influx during the second set of KCl stimulations (**B**); As a negative control, PnPP-19 does not influence calcium influx on its own (**C**).

**Figure 5:**
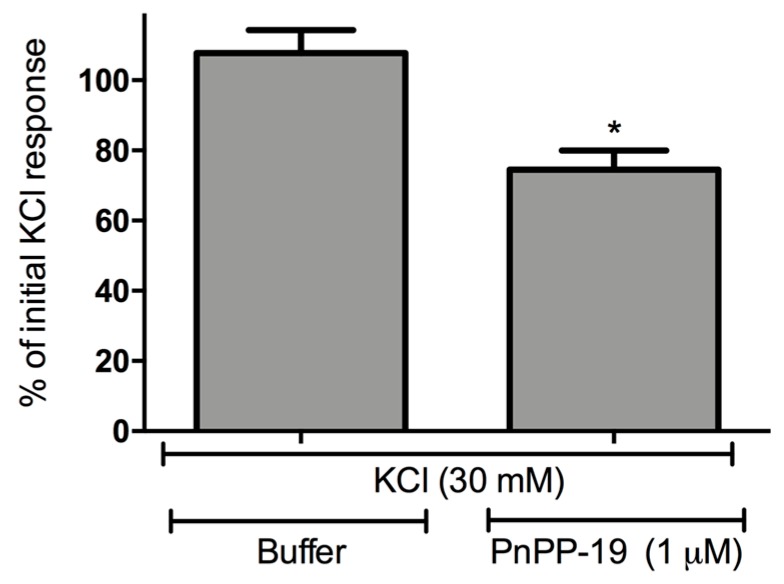
Effect of pre-incubation with PnPP-19 (1 μM) on KCl-evoked (30 mM) responses. The bars represent the percentage of the maximum amplitude response during the second set of KCl stimuli corresponding to the initial KCl stimulations (100%). The peak of response in each situation was calculated, and the amplitude was assessed by diminishing this value of the baseline. The baseline corresponds to the pre-incubation of cells with buffer, before any KCl stimulation. Data shown are the means ± SEM (*n* = 5). * *p* < 0.05 compared with KCl (30 mM) + Buffer (two-tailed *t*-test).

DRG neurons were isolated and the calcium influx was evaluated using fluorescence microscopy. Cell stimulation with KCl (30 mM) induced calcium influx and consequently an increase of intracellular calcium concentration. The perfusion of the cells for 5 min with buffer did not alter the profile of calcium influx induced by KCl during the second course of stimulation if compared with the first set of stimuli (Figure 4 and Figure 5). On the other hand, incubation of DRG neurons with PnPP-19 (1 μM) for 5 min induced a decrease of approximately 20% of calcium influx during the second course of stimulation with KCl. In addition, PnPP-19 (1 μM) did not cause any alteration of intracellular calcium concentration when the cells were not stimulated; therefore, no change of calcium concentration was observed during the 5 min period of cell perfusion with PnPP-19 (Figure 4).

### 2.4. β-Arrestin2 Recruitment Induced by DAMGO and PnPP-19

**Figure 6:**
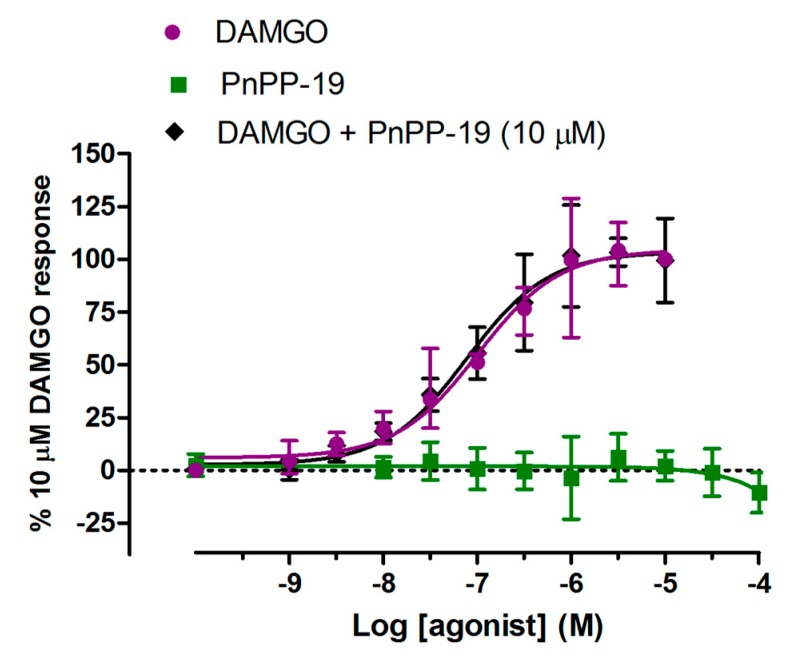
Recruitment of β-arrestin2 by activation of μ-opioid receptors. Stably transfected HEK293 cells coexpressing μ-opioid receptors and β-arrestin2 were pretreated for 60 min with DAMGO or PnPP-19 at the indicated concentrations. The group “DAMGO + PnPP-19” represents prior incubation of the cells with 10 μM of PnPP-19 for 30 min. β-arrestin2 recruitment was quantified by high content imaging complementation assay as described in Materials and Methods (*n* = 5).

Here, we intended to evaluate whether stimulation of HEK293T cells, coexpressing μ-opioid receptor-Yc and β-arrestin2-Yn, by PnPP-19 or the selective μ-opioid agonist [D-Ala^2^, MePhe^4^, Gly-ol^5^] encephalin (DAMGO) would induce recruitment of β-arrestin2 by activating μ-opioid receptors. Incubation of the cells with DAMGO could clearly induce β-arrestin2 recruitment (Figure 6). However, different concentrations of PnPP-19 could not induce any μ-opioid receptor-β-arrestin2 association (Figure 6). In addition, pre-incubation of the cells with 10 μM of PnPP-19 was not able to prevent the binding and activation of μ-opioid receptors by the agonist DAMGO (Figure 6).

## 3. Discussion

Previous literature data, obtained using behavioral tests, suggested that the peripheral and central antinociceptive effect induced by PnPP-19 is partially because of μ- and δ-opioid receptors activation [6,7,8]. Therefore, we verified whether this synthetic peptide could indeed directly bind and activate the different isoforms of opioid receptors (μ-, δ- and κ-). Moreover, since it was already described that activation of opioid receptors suppresses calcium influx through inhibition of different voltage-gated calcium channels, we also investigated the modulation of calcium influx induced by PnPP-19 in DRG neurons. Our data demonstrates that, among the μ-, δ- and κ-opioid receptor subtypes, PnPP-19 may selectively activate, with relatively low potency, the μ-opioid receptor subtype. Remarkably, it seems that the peptide does not induce the recruitment of β-arrestin2 by activating opioid receptors and, most likely, PnPP-19 binds to a different binding site of the opioid receptor than DAMGO (a selective opioid agonist). In addition, PnPP-19 induced an inhibition of calcium channels, very likely through activation of opioid receptors, in the whole-cell patch-clamp assay; it also diminished the calcium influx observed by fluorescence microscopy.

Among all the toxins isolated from the venom of *Phoneutria nigriventer* and its derivatives, only two of them are able to elicit antinociception via activation of opioid receptors. The antinociceptive effect of the toxin PnTx4(6-1), also known as δ-Ctenitoxin-Pn1a [2], is partially blocked when selective antagonists of both μ- and δ-opioid receptors are administered [15]. Likewise, antinociception of PnPP-19, a δ-CNTX-Pn1c derivative [5], occurs also through activation of those very same opioid receptors [6,8]. On the other hand, it was never investigated whether these peptides may directly bind and activate opioid receptors. Our present data show that PnPP-19 might selectively activate, with low potency, only the μ-opioid receptor subtype. However, through behavioral experiments, it was shown that the antinociception induced by PnPP-19 also involves activation of δ-opioid receptors. Here, we demonstrated that PnPP-19 is incapable of activating directly δ-opioid receptors. Therefore, activation of this specific subtype of receptor in vivo may occur via an indirectly pathway, as previously suggested by Freitas and collaborators [6].

PnPP-19 is the first synthetic peptide, derived from a spider toxin, proven to act directly on opioid receptors, and more specifically, on μ-opioid receptor subtype. Novel ligands of the μ-opioid receptor are of clinical and social importance since the common used analgesic drugs, such as morphine, fentanyl and oxymorphone, elicit both their beneficial pharmacological effect and undesirable side effects through activation of opioid receptors [16]. One of the very serious and life-threatening conditions developed following the use of the usual opioid agonist medicines is respiratory paralysis [16,17]. It has been demonstrated that the induction of respiratory paralysis, as well as other side effects, after the use of opioids may be linked with the recruitment of the β-arrestin pathway, which is stimulated downstream following activation of μ-opioid receptor [18,19,20,21,22]. Since opioid receptors are still one of the most relevant targets for pain treatment, great effort is being put in the development of new opioid agonists that elicit fewer negative side effects [22,23]. In this way, the lack of β-arrestin2 recruitment by PnPP-19 underlines the potential of this peptide as a possible lead compound in the development of improved opioid agonists. Recently, a very selective and potent μ-opioid agonist was developed and named PZM21. Despite its great potency and selectiveness against μ-opioid receptors, administration of PZM21 induced minimal β-arrestin2 recruitment. Therefore, the use of PZM21 induced a long-lasting analgesia along with decreased respiratory depression and constipation when compared to morphine [10]. For this reason, studies concerning the exact mechanism of action of PnPP-19 in the pain pathway are of interest since PnPP-19 showed no induction of β-arrestin2 recruitment in cell culture. It thus seems that the peptide has a site of interaction different from the DAMGO binding site, since in our experiments the presence of PnPP-19 has no influence on DAMGO-induced β-arrestin2 recruitment. However, one other hypothesis for the lack of β-arrestin2 recruitment by PnPP-19 could be the low potency of which PnPP-19 might bind to μ-opioid receptors. Therefore, further investigation is required in order to elucidate the exact mechanism of PnPP-19 interaction with the opioid receptors. Moreover, a better characterization of the target of this synthetic peptide in erectile function is required in order to develop a PnPP-19 derived drug without unwanted side effects.

The interaction between opioid receptors and ion channels has been a subject of much interest during decades. Various studies suggest that activation of opioid receptors causes hyperpolarization of the cell and consequently prevents neurotransmitter release by inducing an inhibition of calcium channels [11,24,25] and activation potassium channels [11,26,27,28]. According to in vitro studies, incubation of a selective agonist of μ-opioid receptors with HEK293 cells co-expressing μ-opioid receptors together with voltage-gated N-type calcium channels (Cav2.2) or R-type (Cav2.3) channels induced an inhibition of both calcium channels tested [29]. Moreover, experiments, conducted with primary culture of vestibular afferent neurons and DRG neurons, suggest that selective stimulation of μ-opioid receptors may inhibit T-, L- and N-type calcium channels supposedly through activation of a Gα_i/o_ protein [30,31,32]. Accordingly, our results demonstrate that PnPP-19 inhibits calcium influx in DRG neurons and that this inhibition is suppressed by the unspecific opioid antagonist naloxone. These data show that inhibition of calcium influx induced by PnPP-19 is mediated by activation of opioid receptors. Recently, it was demonstrated that DAMGO (selective μ-opioid agonist) induces inhibition of calcium influx and action potential-evoked Ca^2+^ fluorescent transients in individual peripheral nociceptive fiber free nerve endings from trigeminal ganglion. The authors have shown that activation of “big conductance” Ca^2+^-activated K^+^ channels (BK_Ca_) mediates this inhibition of calcium influx induced by DAMGO. Furthermore, the activation of this subtype of potassium channel plays a major role on μ-opioid induced antinociception in a behavioral test for trigeminal nociception. Therefore, it is likely that PnPP-19 might also modulate potassium channels, since this synthetic peptide may act as an opioid agonist. However, further experiments are needed to investigate whether PnPP-19 indeed interferes with potassium channel activity.

In conclusion, the data we present here shows for the first time a spider toxin derivative that may act as a selective μ-opioid agonist. PnPP-19 directly binds and activates, albeit with low potency, only the μ-opioid receptor subtype. In DRG neurons, the activation of μ-opioid receptors induced by PnPP-19 generates an inhibition of calcium channels, consequently reducing or even eliminating calcium influx. This modulation of calcium channels appears to follow activation of opioid receptors, confirming once again the role of PnPP-19 as an opioid agonist. Interestingly, notwithstanding the activation of opioid receptors, PnPP-19 does not induce β-arrestin2 recruitment. This could be due its low potency; however, it may also be a consequence of a differential opioid activation mechanism in which β-arrestin2 recruitment is not stimulated. Further studies with PnPP-19 could lead to the development of new and more potent opioid agonists that in turn could elicit antinociception with possibly less side effects by not inducing recruitment of β-arrestin2.

## 4. Materials and Methods

### 4.1. Expression of Voltage-Gated Potassium Channels in *Xenopus laevis* Oocytes

*Xenopus laevis* oocytes were isolated as previously described [33]. Oocytes were co-injected with 0.5 ng/50 nL of GIRK1, 0.5 ng/50 nL of GIRK2, and 10 ng/50 nL of RGS4 cRNA, with the addition of 10 ng/50 nL of either hMOR, hKOR, hDOR, hMORW318L, or hMORW318Y/H319Y cRNA. Injected oocytes were maintained in ND-96 solution (composition: 2 mM KCl, 96 mM NaCl, 1 mM MgCl_2_, 1.8 mM CaCl_2,_ 5 mM HEPES, pH 7.5) supplemented with 50 μg/mL of gentamicin sulfate.

### 4.2. Electrophysiological Recordings: *Xenopus laevis* Oocytes

Whole-cell currents from oocytes were recorded from 1 to 2 days after injection using the two-microelectrode voltage-clamp technique. Resistances of voltage and current electrodes were kept between 0.7 and 1.5 MΩ and were filled with 3 M KCl. Currents were filtered at 20 Hz, using a 4-pole low-pass Bessel filter. To eliminate the effect of the voltage drop across the bath-grounding electrode, the bath potential was actively controlled. All experiments were performed at room temperature [19,20,21,22,23]. At the start and the end of each experiment, oocytes were superfused with low-potassium (ND-96) solution (composition: 2 mM KCl, 96 mM NaCl, 1 mM MgCl_2_, 1.8 mM CaCl_2_, 5 mM HEPES, pH 7.5). During application of increasing concentrations of ligands, oocytes were superfused with high-potassium (HK) solution (composition: 96 mM KCl, 2 mM NaCl, 1 mM MgCl_2_, 1.8 mM CaCl_2_, 5 mM HEPES, pH 7.5). In HK solution, the K^+^ equilibrium potential is close to 0 mV and enables K^+^ inward currents to flow through inwardly rectifying K^+^ channels at negative holding potentials. A gravity-controlled fast perfusion system was used to ensure rapid solution exchanges. Analysis of un-injected cells (*n* = 3), under the same experimental conditions as injected oocytes, revealed an endogenous current that amounted maximally 1% as compared with the current measured in injected oocytes. Application of opioid ligands did not evoke an increase of the conductance in un-injected oocytes. In each experiment, oocytes were clamped at a holding potential of −70 mV and super-fused with ND-96 solution. Next, the super-fusion solution was switched from ND-96 to HK solution, after which increasing concentrations of morphine or peptide were applied. Each concentration was applied for as long as needed to achieve a steady-state GIRK1/GIRK2 current activation. Each ligand concentration was washed out by super-fusing it with an HK solution. During this washout period, the channels return to the control current level as a result of a deactivation process that is accelerated dramatically in the presence of RGS4, as previously described [34]. At the end of each experiment, the oocyte was super-fused with HK solution containing 300 μM BaCl_2_, causing a blockage of the net GIRK1/2-gated inward current. Finally, the super-fusion was switched back to ND-96 solution to confirm complete reversibility.

### 4.3. Data Analysis of Two-Microelectrode Voltage-Clamp

The pCLAMP program (Axon Instruments, pCLAMP, Sunnyvale CA, USA) was used for data acquisition, and data files were directly imported, analyzed, and visualized with a custom-made add-in for Microsoft Excel (Redmond, WA, USA). The percentage-activated current was calculated using the equation: percentage activation = activated current amplitude control current amplitude × 100 − 100 and 0% was taken as the control current level. Current percentages were then used for the calculation of concentration–response curves, using the Hill equation I = I_max_/[1 + (EC_50_/A)*^n^*^H^], where I represents the current percentage, I_max_ the maximal current percentage, EC_50_ the concentration of the agonist that evokes the half-maximal response, A the concentration of agonist, and n_H_ the Hill coefficient. Averaged data are indicated as means ± SEM and were calculated using n experiments, where n indicates the number of oocytes tested. For each experiment, the number of oocytes tested was at least 6 (*n* > 6) For each experiment, averaged current percentages were normalized to 100%, and an averaged concentration–response curve was drawn using the average ec_50_ values and Hill coefficients of n experiments. Statistical analysis of differences between groups was carried out with Student’s *t*-test, and a probability of 0.05 was taken as the level of statistical significance.

### 4.4. DRG Culture

DRGs were isolated from adult Wistar rats (200 ± 300 g) and neurons cultured as described by Lindsay (1988) [35] with minor modifications. The neurons were isolated and washed by gravity in phosphate-buffered saline (PBS). The cells were than incubated with collagenase type IV (sigma, St. Louis, MO, USA) solution (5 mL of Dulbecco’s modified Eagle’s medium—DMEM; 10% *v*/*v* fetal bovine serum; penicillin 200 units/mL—streptomycin 200 μg/mL; 12.5 mg of collagenase type IV) for 90 min at 37 °C. After that, ganglions were washed 3 times by gravity in PBS and trypsin solution (2500–6000 BAEE U/ML, sigma, St. Louis, MO, USA) was added. In order to dissociate the DRG neurons, the ganglions were taken up and down with the use of a fine tipped transfer pipette. Cells were then incubated with the trypsin solution for 10 min at 37 °C. After the incubation period, 1 mL of bovine serum albumin (BSA) solution (16% *v*/*v* in PBS) was added and cells were more firmly dissociated. The cell suspension was added on the top of 3 mL BSA solution and centrifuged at 500× *g* for 6 min. The supernatant was discarded and the pellet was resuspended in complete media (DMEM media; 10% *v*/*v* fetal bovine serum; 1% *v*/*v* penicillin/streptomycin; 0.1% *v*/*v* NGF). Cells were plated on poly-l-lysine and laminin coated cover slips and incubated at 37 °C with 5% CO_2_ in a humidified incubator. The study was approved by the local Ethics Committee on Animal Experimentation (CETEA) of UFMG (Protocol number: 233/2013).

### 4.5. Whole-Cell Voltage-Clamp

DRG neurons were used for the measurements after 48 h of cell culture. The calcium current recordings were obtained by using the Patch Clamp amplifiers type EPC-9/EPC-10 (HEKA Instruments, Lambrecht/Pfalz, Germany) and the PULSE/PATCHMASTER data acquisition program (HEKA Instruments, Lambrecht/Pfalz, Germany) adjusted for the Whole Cell Voltage-Clamp configuration. Low resistance patch electrodes (3–4 MΩ) were filled with solution containing (in mM): 130 CsCl, 2.5 MgCl_2_, 10 HEPES, 5 EGTA, 3 Na_2_-ATP and 0.5 Li_3_-GTP, pH 7.4 adjusted with 1 M CsOH. The external/bath solution contained (in mM): 125 CsCl, 10 BaCl_2_, 1 MgCl_2_, 10 HEPES and 60 Glucose, pH 7.4 adjusted with 1 M CsOH. An Ag-AgCl electrode was used as reference. The recordings were filtered with a Bessel low-pass filter set at 2.9 kHz and digitalized at a 10 kHz rate (100 μs interval) through an AD/DA interface (ITC 1600). Capacitive currents were electronically compensated and a P/4 protocol was used to correct the linear leakage current and to subtract residual capacity (BEZANILLA, ARMSTRONG, 1977) [36]. After establishing the Whole Cell configuration, the calcium current was evoked from negative holding potential of −90 mV to 10 mV (200 ms). Once the calcium current showed stable amplitude values, PnPP-19, morphine or naloxone were added separately to the bath solution to give a final concentration of 1 μM, 1 μM and 10 μM, respectively. To test whether the effect of PnPP-19 was through opioid receptors, cells were prior incubated with 10 μM of naloxone for 30 min. The experiments were performed on 35 mm diameter acrylic Petri dishes using inverted microscope (Axiovert 20, Carl Zeiss, Jena, Germany or Nikon TMF-100, Nikon, Chiyoda-Ku, Japan).

### 4.6. Calcium Imaging

The experiments were performed after 24 h of DRG neurons dissociation. On the day of the experiments, cells were incubated with Fura 2-AM (5 mM, 30 min, 37 °C). Intracellular Ca^2+^ concentrations ([Ca^2+^]i) in individual neurons were estimated as the ratios of peak fluorescence intensities (measured at 500 nm) at excitation wavelengths of 340 and 380 nm, respectively (Bundey & Kendall, 1999) [37], using an Improvision imaging system. DRG neurons were superfused (2 mL min^−1^) with buffer (NaCl 145 mM; KCl 5 mM; CaCl_2_ 2 mM; MgSO_4_ 1 mM; HEPES 10 mM; glucose 10 mM) for 1 min followed by three consecutive KCl (30 mM) stimulations. After that, cells were perfused with buffer (control group) or PnPP-19 (1 μM) dissolved in buffer for 5 min, and again depolarized by KCl (30 mM) at three different time points. Representative traces of calcium influx in a single DRG neuron are shown. Results are presented as means ± SEM and indicate the percentage of calcium influx related to the peak of calcium influx during the first course of activation with KCl (100%). Statistical analyses were carried out using GraphPrism software (version 7.0a, GraphPad Software, La Jolla, CA, USA, 2016). Our data were distributed normally and analyzed statistically by two-tailed *t*-test. Probabilities less than 5% (*p* < 0.05) were considered to be statistically significant.

### 4.7. Beta-Arrestin2 Recruitment

HEK293T were cultured in DMEM (Sigma-Aldrich) supplemented with 10% *v*/*v* fetal bovine serum. These cells were coexpressing μ-opioid receptor-Yc and β-arrestin2-Yn (Yc and Yn are complementary fragments of yellow fluorescent protein-YFP). To analyze whether activation of μ-opioid receptor would induce recruitment of β-arrestin2, the Bimolecular fluorescence complementation (BiFC) based detection of μ-opioid receptor-β-arrestin2 association was conducted. The cells were seeded at 33,000 cells/well onto poly (d-lysine)-coated Greiner 655,090 imaging plates. Plates were kept in a humidified incubator at 37 °C filled with 5% CO_2_ for 24 h. HEK293T were stimulated with the selective opioid agonist DAMGO (Tocris, Minneapolis, MN, USA) or the synthetic peptide PnPP-19 in HEPES-buffered saline solution (HBSS) including 0.1% *v*/*v* BSA (10^−10^ M–10^−4^ M) for 60 min at 37 °C. In the experiment where we investigated whether PnPP-19 could impair the binding of DAMGO to μ-opioid receptors, cells were preincubated with PnPP-19 10 μM (30 min, 37 °C). After that, cells were fixated with 3% paraformaldehyde in PBS for 10 min at room temperature. Then, cells were washed once with PBS and the cell nuclei were stained for 15 min with H33342 (2 μg/mL in PBS, Sigma, St. Louis, MO, USA). H33342 was then removed by a final PBS wash. Images (4 central sites/well) were acquired automatically on the IX Ultra confocal plate reader, using 405 nm/488 nm laser lines for H33342 and complemented YFP excitation, respectively. Data was analyzed by the use of MetaXpress software (version 5.3, Sunnyvale, CA, USA, 2013) as described by Liu and co-authors [38] and normalized by 10 μM of DAMGO (100%).

## Supplementary Materials

The following are available online at www.mdpi.com/2072-6651/10/1/43/s1, Figure S1: Representative current traces evoked from *X. laevis* oocytes co-expressing GIRK1/GIRK2 channels and RGS4. PnPP-19 does not interact with GIRK channels; Figure S2: Representative current traces evoked from *X. laevis* oocytes co-expressing GIRK1/GIRK2 channels and RGS4 with hMOR.
