# Supplementary material for: The Peptide PnPP-19, a Spider Toxin Derivative, Activates μ-Opioid Receptors and Modulates Calcium Channels
Source: Toxins (Basel). 2018 Jan 15;10(1):43. doi: 10.3390/toxins10010043 (PMC5793130; doi:10.3390/toxins10010043)
Supplement: Supplementary file 1 [file toxins-10-00043-s001.pdf]

Supplementary Materials: The Peptide PnPP-19, A Spider Toxin Derivative, Activates μ-Opioid Receptors and Modulates Calcium Channels

Ana C N Freitas, Steve Peigneur, Flávio H P Macedo, José E. Menezes-Filho, Paul Millns, Liciane F. Medeiros, Maria A. Arruda, Jader Cruz, Nicholas D. Holliday Jan Tytgat, Gareth Hathway and Maria Elena de Lima

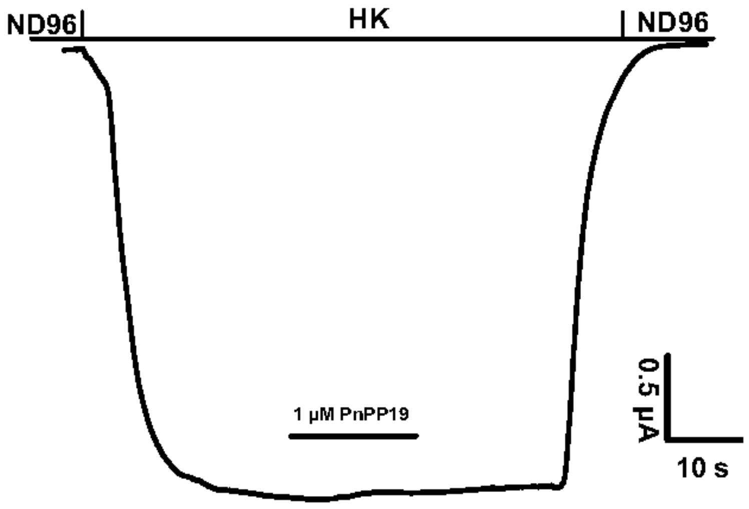

Figure S1. Representative current traces evoked from *X. laevis* oocytes co-expressing GIRK1/GIRK2 channels and RGS4. PnPP19 does not interact with GIRK channels.

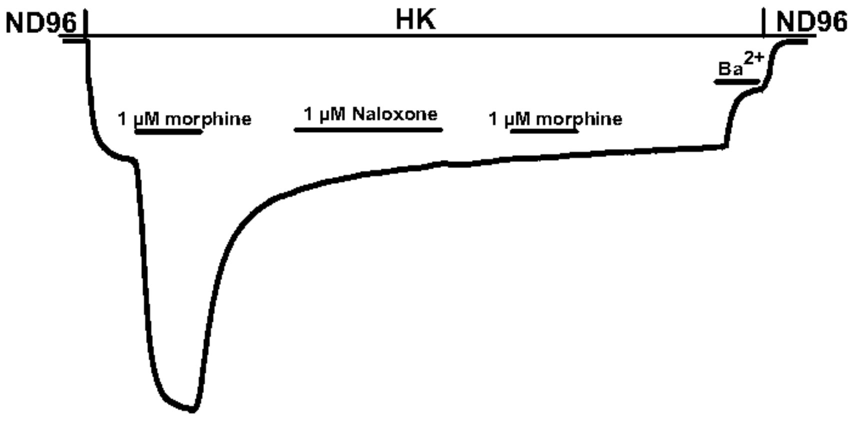

Figure S2. Representative current traces evoked from *X. laevis* oocytes co-expressing GIRK1/GIRK2 channels and RGS4 with hMOR. In addition, 1 μM naloxone inhibits the agonistic activity of 1 μM morphine.
